# SIRT3 a Major Player in Attenuation of Hepatic Ischemia-Reperfusion Injury by Reducing ROS via Its Downstream Mediators: SOD2, CYP-D, and HIF-1*α*

**DOI:** 10.1155/2018/2976957

**Published:** 2018-11-13

**Authors:** Gaurav Katwal, Dilip Baral, Xiaoli Fan, He Weiyang, Xinjiang Zhang, Li Ling, Yan Xiong, Qifa Ye, Yanfeng Wang

**Affiliations:** ^1^Zhongnan Hospital of Wuhan University, Institute of Hepatobiliary Diseases of Wuhan University, Transplant Center of Wuhan University, Hubei Key Laboratory of Medical Technology on Transplantation, Wuhan, Hubei 430071, China; ^2^The 3rd Xiangya Hospital of Central South University, Research Center of National Health Ministry on Transplantation Medicine Engineering and Technology, Changsha 410013, China

## Abstract

Reactive oxygen species (ROS) production in hepatic ischemia-reperfusion injury (IRI) is a complex process where multiple cellular and molecular pathways are involved. Few of those molecular pathways are under the direct influence of SIRT3 and its downstream mediators. SIRT3 plays a major role in the mechanism of IRI, and its activation has been shown to attenuate the deleterious effect of ROS during IRI via SOD2-, CYP-D-, and HIF-1*α*-mediated pathways. The objective of this review is to analyze the current knowledge on SIRT3 and its downstream mediators: SOD2, CYP-D, and HIF-1*α*, and their role in IRI. For the references of this review article, we have searched the bibliographic databases of PubMed, Web of Science databases, MEDLINE, and EMBASE with the headings “SIRT3,” “SOD2,” “CYP-D,” “HIF-1*α*,” and “liver IRI.” Priority was given to recent experimental articles that provide information on ROS modulation by these proteins. All the recent advancement demonstrates that activation of SIRT3 can suppress ROS production during IRI through various pathways and few of those are via SOD2, CYP-D, and HIF-1*α*. This effect can improve the quality of the remnant liver following resection as well as a transplanted liver. More research is warranted to disclose its role in IRI attenuation via this pathway.

## 1. Introduction

Ischemia is tissue hypoxia as a result of the loss of the arterial perfusion or the loss of the venous drainage of the organ. After the restoration of the circulation following liver resection and transplant surgery, high levels of ROS are produced due to mitochondrial dysfunction [1]. According to the concept of mitohormesis, these organelles signal through the generation of ROS that are produced by the electron transport chain during normal physiological stress and help for the adaptation [2]. But ROS that are produced during liver resection and transplantation are pathological. And these can damage DNA as well as other cellular macromolecules like proteins and lipids leading to inflammation, congestion, necrosis, and apoptosis [3, 4]. A timely intervention that removes these abnormal and dysfunctional mitochondria not only improves the function but also promotes the tissue survival [4, 5]. One of such mechanisms is mitophagy, which is essential for the control of the vicious cycle of ROS production [1, 6, 7]. Likewise, Galaris et al. [8] have brilliantly described strategies to reduce these harmful ROS (a) to potentiate the endogenous antioxidant capacity, (b) to scavenge reactive species, and (c) to inhibit the formation of ROS. The purpose of this review is to focus on the current knowledge on SIRT3 and its downstream mediators, namely, SOD2, CypD, and HIF-1*α* and their impact on hepatic IRI via attenuation of ROS.

During IRI, multiple signaling pathways are activated by ROS. And the crosstalk within the cells is regulated by different molecules. One of such molecules regarded as the master regulator of the mitochondrial metabolism is sirtuin, which is also an important energy sensor [9–12]. The mammalian sirtuins are evolutionally highly conserved class III histone deacetylases protein with nicotinamide-adenine dinucleotide- (NAD+-) dependent deacetylase activities and/or ADP-ribosyltransferase activities [13–15]. There are seven members in the human sirtuin family: SIRT1–7. SIRT1 and SIRT6 have deacetylase activities as well as relatively weak ADP-ribosyltransferase activities whereas SIRT4 has only ADP-ribosyltransferase activity [14]. Sirtuins are located in the different compartments of the cell, such as the cytoplasmic (SIRT1 and 2), the nuclear (SIRT1, 6, and 7), and the mitochondrial (SIRT3, 4, and 5), respectively [5, 6].

### 1.1. Homeostasis and SIRT3

Sirtuins carry out the function of deacetylation of target proteins: acetyl-CoA acetyl-transferase-1, acetyl-CoA synthase-2, long-chain acyl-CoA dehydrogenase (LCAD) [16, 17], glutamate dehydrogenase, 3-hydroxy-3-methylglutaryl CoA synthase-2, isocitrate dehydrogenase-2, pyruvate dehydrogenase [18], ornithine transcarbamoylase [19], and manganese superoxide dismutase (MnSOD) [20]. All of these are the intermediate products of lipid, glucose, and protein metabolism whereas MnSOD is a potent mitochondrial antioxidant. Other than this, SIRT3 also deacetylates components of electron transport chain complexes like NDUFA9, ATP synthases, succinate dehydrogenase, and flavoprotein [1, 14, 15, 21, 22]. SIRT3 also enhances the activities of the adenosine monophosphate-activated protein kinase (AMPK) and peroxisome proliferator-activated receptor gamma coactivator 1-alpha (PGC1*α*) and increases the daf-16 homolog FOXO3a-dependent gene expressions. These complex proteins also actively take part in lipid, protein, and glucose metabolism along with the regulation of apoptosis by FOXO3a and thus regulate mitochondrial functions through indirect mechanisms also [23].

The gene of human SIRT3 is located within the 11p15.5 chromosomal region, and variations in the gene of SIRT3 have been shown to prolong human lifespan. The full-length SIRT3 protein is 44 kDa and contains 399 residues with an N-terminal mitochondrial targeting sequence and is enzymatically inactive, which is processed to produce an active 28 kDa polypeptide by cleaving the N-terminal 101 residue upon import into the mitochondrial matrix [24–28]. SIRT3 plays a major role in the mitochondrial energy production and the metabolic homeostasis [22]. NAD has a critical role in ATP generation in the mitochondria, and it is an electron carrier. Increased NAD+ levels trigger a regulatory pathway that activates SIRT3 and leads to the deacetylation of specific targets. And thus, Onyango et al. [29] proposed that SIRT3 has a critical role in sensing NAD+ level or NAD+/NADH ratios. The expression of SIRT3 mRNA is found to be increased during low energy input such as in intermittent fasting [30] and caloric restriction (CR) [31]. This modulates the mitochondria to adapt to low energy input, by promoting amino acid catabolism, b-oxidation, acetate recycling, antioxidant defense, and thus oxidative phosphorylation and energy production within the mitochondria [32]. In a normal fasted liver, Liu et al. [33] also found that greater OXPHOS capacity was achieved by SIRT3-mediated deacetylation of LRP130. High-fat diet (HFD) with saturated fatty acids (SFAs) was found to activate SIRT3 expression initially but, in chronic HFD, led to the suppression of SIRT3. Overexpression of SIRT3 protected hepatocytes from lipotoxicity-induced cell death and was found to be protective against nonalcoholic fatty liver disease (NAFLD) [34], whereas suppression of SIRT3 led to the increase in mitochondrial protein acetylation and further increased hepatocyte susceptibility to lipotoxicity-induced cell death [34, 35].

### 1.2. ROS Generation and Its Attenuation by SOD2

There are 3 forms of SOD: cytosolic copper-zinc-dependent (Cu-Zn-SOD/SOD1), mitochondrial manganese-dependent SOD (MnSOD/SOD2), and the extracellular copper-zinc-dependent form extracellular SOD (ecSOD/SOD3) [36]. Our focus is on SOD2 due to its mitochondrial location and its key role in ROS attenuation during IRI. MnSOD is an old, evolutionarily conserved protein present in almost all species, and MnSOD lysine is an acetylation target in the regulation of its enzymatic activity [20]. The gene of MnSOD is located on the 6q25.3 region of the sixth chromosome in humans that encodes a ~223 amino acid which is a 26 kDa precursor monomer. Upon import into the mitochondria, it gets processed into a 22 kDa monomer and incorporates an Mn + 3 ion to form an 88 kDa homotetramer [37]. It is the primary mitochondrial scavenging enzyme that converts superoxide to hydrogen peroxide, which is finally converted to water by catalase [13, 18, 19].

Several mechanisms have been shown to contribute to ROS production, but hypoxia/hypoxic periods that senses the oxygen tension play a dominant role in the activation of ROS-producing enzyme systems. And this concept of hypoxia-induced production of ROS can be easily applied to the transplant settings where notoriously hypoxia-damaged donor organs are used [38]. In postischemic tissues, three hypoxia-sensing systems are the main source of ROS production, namely, the mitochondrial electron transport chain-associated enzymes (mainly through complex I and complex III), xanthine oxidase, and reduced form of nicotinamide adenine dinucleotide phosphate oxidases as well as uncoupled nitric oxide synthase [8, 39–41]. In the case of the solid organ transplantation, ROS at first is generated from the vascular cells of the donor organ following reperfusion. And the second burst of ROS is from phagocytic cells of the recipient. After the reperfusion, the neutrophils and the macrophages adhere to the endothelium where they get activated by ROS from the vascular cells [38]. Thus, ROS is a double-edged sword, and in a low concentration, they function as a second messenger and facilitate innate immune system signaling pathways to full functionality, whereas in a higher concentration, they operate as a noxious molecule that activates the innate immune system against the cause that might activate a cell death pathway.

**Figure 1:**
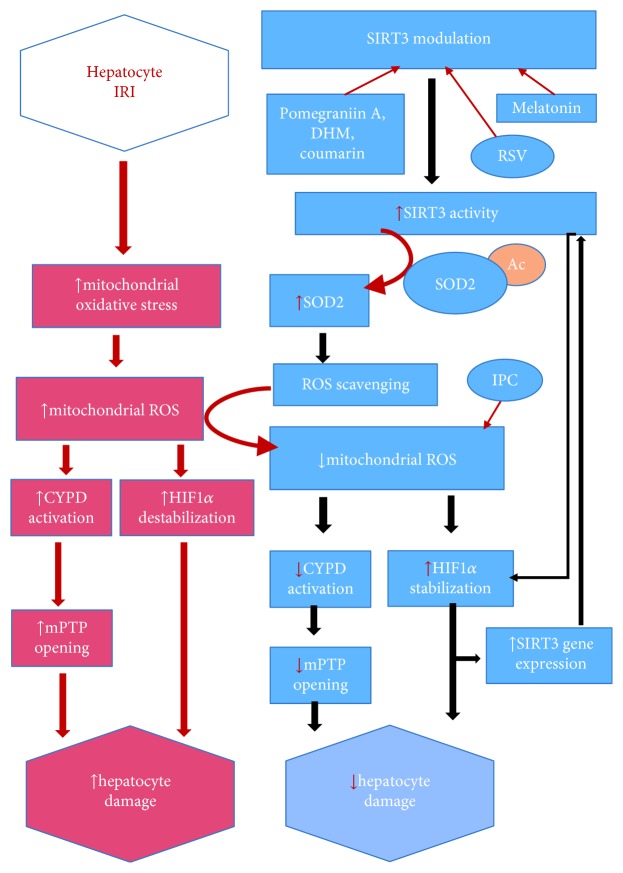
Increased activity of Sirt3 promotes deacetylation of SOD2. Increased activity of Sirt3 promotes deacetylation of SOD2; this reduces the cellular oxidative stress via ROS scavenging. Sirt3 also stabilizes HIF1*α*, which activates Sirt3 gene promoter that leads to increased synthesis of Sirt3 mRNA transcripts, and deactivates CYPD and subsequently decreases mPTP opening. All these processes orchestrate to reduce hepatocyte damage. Dihydromyricetin (DHM), ischemic preconditioning (IPC), and Resveratrol (RSV).

Liu et al. [42] have demonstrated that hepatocytes with the decreased level of SIRT3 are prone to ROS injury in vitro and in vivo and verified that SIRT3 boosts ROS scavenging, improves mitochondrial biogenesis, and prevents mitochondrial fragmentation from ROS (see Figure 1). Calorie restriction can also provoke the SIRT3 and leads to the reduction of ROS via SOD2. SOD2 expression alone can only moderately reduce cellular ROS whereas SIRT3-mediated deacetylation can significantly boost the ability of SOD2 and ultimately reduce the cellular ROS [31]. An experiment with HepG2 cells under prolonged hyperglycemic stimulation which imitated oxidative stress conditions has shown increased expression of SIRT3, PGC-1a, pCREB, and mitochondrial antioxidant (SOD2, GPx1, and UCP2). And these proteins and enzymes were reduced with the inhibition of SIRT3 [43]. Other mechanisms by which ROS can be decreased are by targeted antioxidant therapy. Mitochondrially targeted antioxidants (MitoQ/Mito-CP) were found to attenuate ROS/RNS and thus subsequently mitochondrial dysfunction in vivo under pathological conditions. It might be due to the attenuation of lipid peroxidation and/or by quenching peroxynitrite. So these properties of antioxidants might have a future therapeutic potential [44]. The potential role of sirtuin (SIRT1/3) activators on different modes of graft preservation that could preserve mitochondrial function and reduce ROS has been explicitly reviewed by Bejaoui et al. [45]. One of such sirtuin activators with the promising result is Resveratrol (RSV) [46]. Data of Gedik et al.'s [47] study indicated that Resveratrol provides liver protection during IRI as early as 45 min of RSV reperfusion compared to that of Hassan-Khabbar et al. [48] where trans-RSV was reperfused for 3 h post-IRI. These studies do prove that RSV is a hepatoprotective agent. Interestingly, RSV was also found to exert antiangiogenic effects by inhibiting HIF-1*α* and VEGF during hepatic IRI [49].

Other than RSV, activation of SOD2 by SIRT3 via pomegranate-derived polyphenols (pomegraniin A) in Caco-2 cells resulted in decreased ROS burden. So pomegraniin A might have the molecular basis for antioxidant therapy in the future [50]. Melatonin, when administered in a therapeutic dose, was found to offer an antioxidant and anti-inflammatory effect in the hepatotoxic model via the AMPK-SIRT3-SOD2 axis [51]. Flavonoid—dihydromyricetin (DHM)—via SIRT3-dependent mechanism also improved mitochondrial respiratory capacity and redox homeostasis in hepatocytes by restoring SOD2 activity [52]. Similarly, 7-hydroxy-3-(4′-methoxyphenyl) coumarin was found to activate SIRT3 and promoted mitochondrial SOD2 activity [53].

Considering all these, SIRT3 is a potential target in the management of the oxidative stress during liver surgeries. And based on these facts, we can conclude that SOD2, a downstream mediator of SIRT3, protects nuclear and mitochondrial DNA as well as other cellular macromolecules from ROS-related damage by its attenuation [14, 15, 24].

### 1.3. SIRT3 Inhibits mPTP Opening via CYP-D

Cyclophilin was first discovered in the year of 1984 as a cytosolic target of CsA. Cyclophilins are considered an evolutionarily conserved peptidyl-prolyl cis-trans isomerase (PPIase) activity [54, 55]. There are 7 major cyclophilins in humans—hCypA (also called hCyp-18a, where 18 is a molecular mass), hCypB (also called hCyp-22/p, 22 kDa), hCypC, hCypD, hCypE, hCyp40 (40 kDa), and hCypNK (first identified from human natural killer cells)—and a total of 16 unique proteins [56]. Human CypD is encoded by peptide-prolyl isomerase F (PPIF) gene and is 207 amino acid long with an N85% homology to its rodent counterparts. After posttranslational modification, matured CypD protein is ~18 kDa that localizes in the mitochondrial matrix [54], whereas CypA and Cyp40 are cytosolic; CypB and CypC due to amino-terminal signal sequences target the ER protein secretory pathway; CypE has an amino-terminal RNA-binding domain and localizes in the nucleus; and CypNK is the largest cyclophilin with hydrophilic and positively charged carboxyl terminus that localizes in the cytoplasm [56].

**Figure 2:**
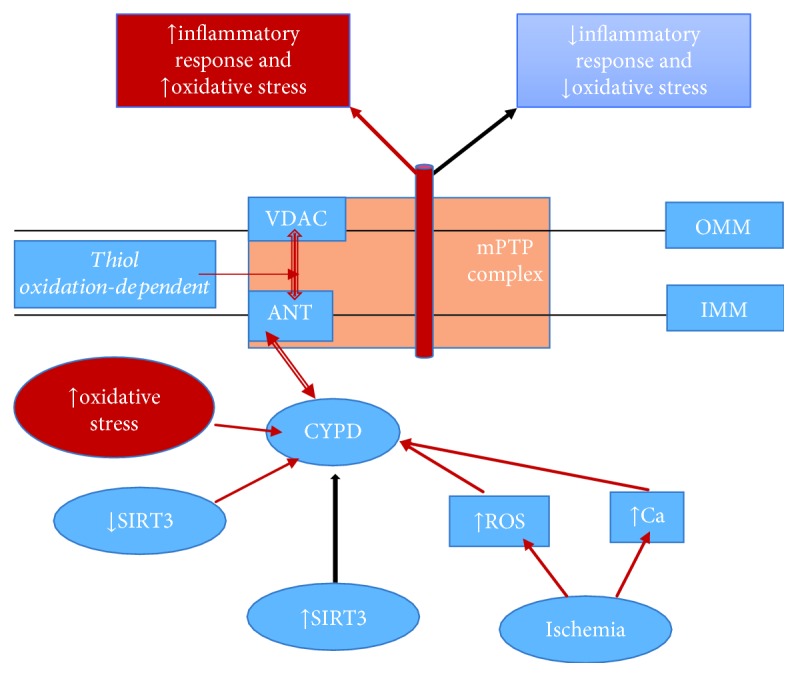
Illustration of adopted and simplified mPTP complex [53]. Increased mitochondrial oxidative stress primes CypD to bind with ANT (thiol oxidation-dependent), and VDAC-ANT complex causes mPTP opening. Increased activities of Sirt3 inhibit CypD-ANT bond and subsequently suppress mPTP opening. For detailed mPTP complex structure please refer to Rizwan, M. et al. [53]

mPTP is a voltage-gated Ca2+-dependent, CsA-sensitive, and high-conductance channel. Its opening causes a sudden increase in inner mitochondrial membrane (IMM) permeability to solute with molecular masses up to 1500 Da [54, 55]. The channel spans between the IMM and the OMM (outer mitochondrial membrane). On the basis of existing data, the fundamental unit of mPTP on IMM consists of ATP synthase F0 domain, which may form a core with ANT, PiC, CKMT1, CypD, and p53 as the peripheral regulatory components (Figure 2). GSK3-*β* and SIRT3 also regulate mPTP indirectly via phosphorylation and acetylation of either regulatory or core components, respectively. On the OMM, mPTP consists of VDAC-HKII and BAK-BAX protein complexes, in addition to their association with OMM permeabilization [54]. CypD binds to ANT and VDAC-ANT complexes; this association is augmented by oxidation of distinct thiol species in ANT molecule during oxidative stress. Thus, the increase in the oxidative stress could increase the mPTP opening by enhancing the thiol oxidation-dependent formation of CypD-ANT complexes in the IMM [54, 57]. Indirubin was found to prevent CypD phosphorylation via inhibition of GSK-3*β*, which led to the inhibition of CypD-ANT interaction and prevented mPTP opening. Furthermore, in NAD+-pretreated animals, mPTP threshold was found to increase due to increased deacetylation of CypD by SIRT3 [58].

Experimental data of Matas et al. [59] showed that increased abundance of CypD in IMM could increase mitochondrial vulnerability to stress. This may be an important factor that could initiate the vicious circle of cellular dysfunction and finally triggers the cell death pathway. Although mPTP formation might be caused by ischemia in the absence of reperfusion, the study with pharmacological and biochemical approaches indicated that mPTP formation occurs mostly at the beginning of reperfusion [60]. Consequently, in postischemic reperfusion, status ROS accumulates, pH normalizes, and rise in Ca2+ creates a perfect condition for mPTP formation [60, 61]. At first, this was demonstrated by Griffiths and Halestrap [62, 63] using Langendorff perfused heart. This phenomenon of calcium-induced loss of membrane potential and OXPHOS that led to the release of proapoptotic protein cytochrome c in the human brain and the liver mitochondria was later demonstrated by Hansson et al. [64] Similarly, CypD^−/−^ mice have shown inhibition of mPTP formation and prevented ATP depletion, subsequently reducing necrotic cell death following renal ischemia/oxidative stress compared to wild-type mice [65]. These results indicated that besides apoptosis, CypD also plays a role in the necroptosis and the autophagy. Interested readers can find elegant literature on the necrotic cell death pathway regulated via CypD reviewed somewhere else [66].

In an experiment with high-fat diet (HFD) condition, liver-specific CypD gene KO resulted in increased glucose intolerance due to excessive hepatic glucose production. This study shows that CypD is a key regulator of mPTP in the liver and it has a critical role in the metabolism under HFD conditions [67]. A crucial role of CypD in triglyceride (TG) metabolism and hepatic steatosis has also been found. Along with excess mPTP opening, CypD stimulation led to the activation of the Ca2+/p38 MAPK/IRE1*α*/SREBP-1c signaling pathway (MAPK (mitogen-activated protein kinase), SREBP (sterol regulatory element-binding protein)) which increased hepatic steatosis [68].

Contrary to all these findings, Parodi-Rullán recently found that SIRT3^−/−^ mice were vulnerable to IRI due to high ROS and mitochondrial protein oxidation with low postischemic recovery. But there was no difference on Cyp-D acetylation level, ROS production, and protein oxidation. And they purposed a compensatory mechanism of increased expression of SIRT4, which was found to reduce apoptosis in H9c2 cells during hypoxia-reoxygenation [69]. Although the precise physiological role of CypD still remains elusive, it is a novel protein with a great potential in the field of biomedical research in the mitochondrial-dependent cell death pathways of cardiovascular [54, 59, 70] liver [71], kidney [65, 72], and brain [73] pathologies along with its involvement in drug resistance in cancer. Taken together, we can state that directly modulating CypD or via SIRT3, modulation of components of mPTP provides protection against IRI by decreasing ROS.

### 1.4. HIF-1*α* Stabilization via SIRT3 Modulates IRI

The detailed analysis of the molecular mechanisms by which HIF-1 activity is regulated and its role in mammalian oxygen homeostasis was given by Wang et al. [74]. The hypoxia-induced factor is a heterodimeric protein that comprises two basic helix-loop-helix proteins containing a PAS domain, with an oxygen-sensitive 120-kDa *α*-subunit (HIF-1*α*) and a constitutively expressed 91 to 94-kDa *β*-subunit, the aryl hydrocarbon nuclear translocator (ARNT or HIF-1*β*) [74]. The *α*-subunit of HIF-1 at a normoxic state is subjected to hydroxylation on proline residue 402 and/or 564 by prolyl hydroxylase domain (PHD) proteins (principally PHD2). It uses O_2_ and *α*-ketoglutarate as substrates to catalyze a dioxygenase reaction. In this reaction, one oxygen atom is inserted into the proline residue and the other into the *α*-ketoglutarate and finally releases succinate and CO2 [10, 75]. But at the hypoxic state, due to the substrate (O2) deficiency and/or the production of ROS from the Qo-site of complex III in the mitochondria, the proline hydroxylase is inhibited due to the oxidation of ferrous ion at the catalytic site. The loss of hydroxylase activity increases HIF-1*α* stability and transactivation function, leading to its dimerization with HIF-1*β*. And then it translocates into the nucleus where HIF-1 binds to its recognition sequence 5′-(A/G)CGTG-3′ in a target gene and thus increases transcription of target gene sequences into mRNA. HIF-1*α* then targets multiple transcription genes that promote aerobic glycolysis, angiogenesis, and metastasis and inhibits the TCA cycle [10, 76].

Schaffner et al. [77] demonstrated that expression of OSTa-OSTb, a bile acid efflux transporters, was modulated by elevated bile acid and hypoxia. In conclusion, OSTa-OSTb induction via HIF-1*α* can protect hepatocyte from the intracellular accumulation of bile salts and hypoxic injury during IRI. Similarly, Ke et al. [78] in their experiment with Keap1-Nrf2 complex (Keap1 is a negative regulator of the Nrf2 pathway) in oxidative injury in IR-stressed OLTs model found that HIF-1-mediated overexpression of HO-1/cyclinD1 protected hepatocytes by reducing inflammation and necrosis/apoptosis via a PI3K-dependent manner. Nitric oxide (NO) modulation by Institute Georges Lopez preservation solution (IGL-1) led to the stabilization of HIF-1*α* and upregulation of heme oxygenase-1 (HO-1), which was found to be protective against cold IRI during preservation particularly in moderately steatotic livers [79]. Similarly, donor rat liver graft pretreated with Mangafodipir (MnDPDP) protected the liver from cold ischemia/reperfusion injury via activation of Nfr2 and HIF-1*α* pathways by increasing catalase and HO-1 activities [80]. Recently, Panisello-Roselló et al. [81, 82] also demonstrated IGL-1 protected the fatty liver against cold IRI via activation of AMPK, reduction of p-TOR/mTOR, stabilization of the cellular ATP content, and high e-NOS expression. These results provide evidence that IGL-1 is a better preservation solution than the University of Wisconsin solution (UW) and histidine-tryptophan-ketoglutarate (HTK) solution.

**Figure 3:**
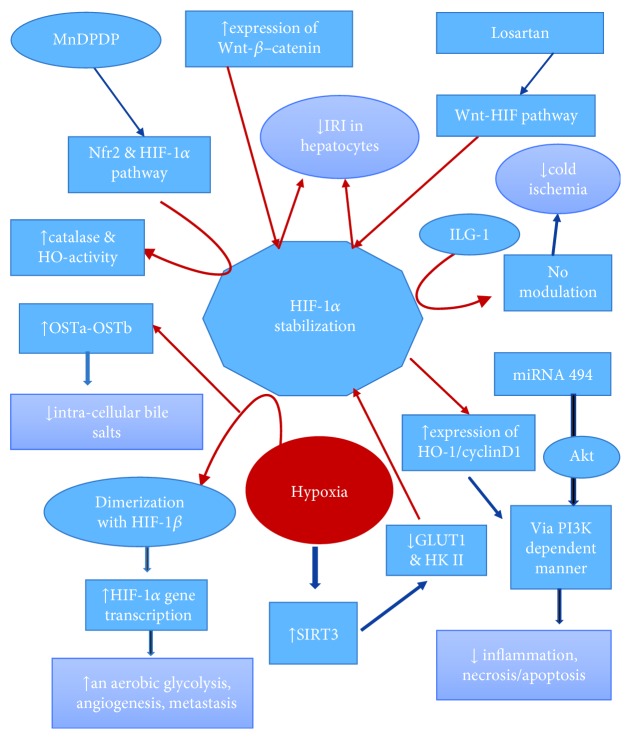
Illustration of HIF-1*α* stabilization via different pathways that finally leads to a decrease in inflammation, necrosis/apoptosis, and IRI of hepatocytes. HIF-1*α* stabilization also leads to an increase in aerobic glycolysis, angiogenesis, and metastasis, a sequel of tumorigenesis. Please find detail explanation in HIF-1*α* Stabilization via SIRT3 Modulates IRI. Heme oxygenase-1 (HO-1), Institute Georges Lopez preservation solution (IGL-1), mangafodipir (MnDPDP), and nitric oxide (NO).

HIF-1*α* stabilization by overexpression of the Wnt–*β*-catenin signaling pathway was found to decrease IRI in hepatocytes [83]. Similarly, the activation of the Wnt-HIF axis with the use of losartan was found to salvage steatotic liver grafts from IRI during transplantation [84]. Stabilization of HIF-1*α* by pretreatment with dimethyloxalylglycine (DMOG) led to the inhibition of iNOS and subsequently protected the kidney following IRI [85]. Normoxic stabilization of HIF-1 was found to protect the cardiac cells against IRI by preventing mPTP opening which reprograms basal cell metabolism from OXPHOS to aerobic glycolysis, and also mitochondrial HKII is partially required for HIF-1 in maintaining mitochondrial integrity [86]. Furthermore, Finley et al. [87] demonstrated that during hypoxia, SIRT3 overexpression blunted the induction of GLUT1 and HKII and revealed that SIRT3 directly regulates the stabilization of HIF-1*α*. SIRT3 also indirectly inhibits HIF-1*α* activity through ROS reduction and that inhibits not only the stabilization but also the activation cascade of HIF-1*α* [10, 87]. Taken together, ROS reduction and HIF-1*α* stabilization harmonize aerobic glucose consumption and act as a tumor promoter [10]. Expression of the HIF-1*α* target gene by liver epithelial cells (LECs) was found to protect the liver from lethal insults like hemorrhagic shock and ischemia-reperfusion probably by paracrine signaling of stromal cell-derived factor-1 (SDF-1) as well as differentiation into parenchymal cells [88]. Similarly, Sun et al. [89] by overexpressing miR-494 in the human liver cell line L02 found augmented HIF-1*α* expression through the PI3K/Akt pathway. And they suggested that miR-494 can be a potential target for hepatic IRI. Considering all this, stabilization of HIF-1*α* seems to promote angiogenesis, tumorigenesis, and glycolysis in the cancer tissue. But in the case of IRI post liver resection or transplant surgery, stabilization of HIF-1*α* directly with pharmacological agents or via modulation of SIRT3 seems to be a futuristic therapeutic option to protect the liver against IRI (Figure 3).

### 1.5. Ischemic Preconditioning (IPC)

**Figure 4:**
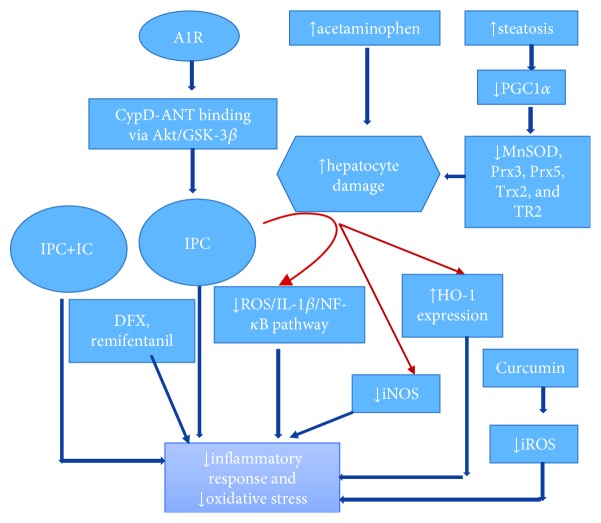
Illustration of surgical and pharmacological Ischemic preconditioning (IPC) via multiple pathways that modulates inflammation and reduces oxidative stress by decreasing mitochondrial ROS. Please find detail explanation in Ischemic Preconditioning (IPC). Adenosine A1 receptors (A1R), deferoxamine (DFX), heme oxygenase-1 (HO-1), intermittent clamping (IC), and induced nitric oxide synthetase (iNOS).

IPC surgically and pharmacologically also has shown exciting results in experimental animal models. IPC modulates by decreasing cellular inflammatory response and oxidant stress [3, 90]. Duarte et al. [91] provided direct evidence of adenosine A1 receptors (A1R) for their ability to preserve mitochondrial function upon pharmacological hepatic preconditioning by modulation of CypD-ANT binding via the Akt/GSK-3*β* pathway. And they suggested that A1R can be used to boost the quality of the donor's liver. Honokiol [92], a natural lignin, is found to protect hepatocytes via activation of SIRT3. It promoted PGC1*α* level and attenuated t-BHP-0mediated mitochondrial fragmentation through the Ku70 dynamin-related protein 1 axis. Similarly, curcumin [93] reduced ROS and decreased IRI in an orthotopic mouse liver transplantation model. It activated peroxisome proliferator-activated receptor *γ* (PPAR*γ*) and inhibited the NF-*κ*b pathway in KCs. By targeting PGC1*α* with agonist (WY-14643), Pantazi et al. [94] found decreased hepatic injury and MDA levels (a measure of lipid peroxidation) in obese rats, enhanced SIRT1 activity with no effect on SIRT1/3 protein expression, enhanced NAD+ levels, augmented ATP levels, and decreased endoplasmic reticulum stress. In another study, remote ischemic conditioning was found to be protective against acetaminophen-induced liver injury by inhibiting the ROS/IL-1*β*/NF-*κ*B pathway and iNOS and increase of HO-1 expression in mice [95]. Sánchez-Ramos et al. [96] in their animal model found that hepatic steatosis downregulates PGC1*α* (an important mediator of IPC) and subsequently decreases the expression of antioxidants (MnSOD, Prx3, Prx5, Trx2, and TR2). This could be the reason steatotic livers have elevated levels of ROS/RNS, which exerts a plethora of damaging effects with an exaggerated reaction to IRI [96, 97]. Recently, preconditioning with Remifentanil, Zhao et al. [98] demonstrated that it could alleviate the hepatic apoptosis and protect the liver against IRI. IRI injury in a porcine hepatectomy model by Trogadas et al. [99] showed both IPC and deferoxamine (DFX) were linked with lower intracranial pressure, attenuation of hepatic IRI, amelioration of the hepatocellular necrosis, apoptosis, and degenerations (Figure 4).

A systemic review of pharmacological strategy and the liver IRI has suggested that the multifactorial and pleiotropic approaches should be considered, respectively, along with categorization of the agents in the rat LT models [100]. But these results have still not been effectively translated in human settings. Similarly, a pilot study from Mexico did not show any effect on the postoperative outcome and had no harmful effects on graft function, morbidity, or mortality rates following 10 min of IPC [101]. Also, a meta-analysis of 11 RCT that included 699 liver resection patients showed early protection by reducing ROS production. But there were no long-term statistical differences in morbidity and mortality [102]. In the case of liver resection, intermittent clamping (IC) along with IPC (IPC + IC) compared to continuous clamping (CC) alone was associated with a reduction in liver failure, postoperative morbidity, ICU stays, blood loss volume, prothrombin time, or aminotransferase levels [103]. Similar results of early benefits but no long-term statistical significance were obtained in LDLT [104] and recently in a meta-analysis by Robertson et al. [105]. Based on the above considerations, it is reasonable to say that the ideal approach to reducing oxidative stress from IRI should be an early and effective removal of excess ROS. Along with its downstream mediators, SIRT3 is a major role player in ROS suppression. And modulation of these proteins can improve liver function following IRI and influences short- and long-term outcomes. The interested reader can find an explicit explanation on the pathophysiology of ROS and its attenuation via pharmacotherapy [106, 107], ischemic preconditioning (IPC) [107], mitophagy [1, 6, 7], autophagy in transplantation [108], ROS, and its emerging role in immunity [109] somewhere else. We could not cover these topics due to the space limitation.

### 1.6. Is SIRT3 Good or Bad?

Finally, to make the balance discussion on Sirt3, without the discussion on detrimental effects of Sirt3, the review will be incomplete. The role of SIRT3 in neoplasia is cell-type specific and potentially quite complex. Otto Warburg first noted that cancer cells shift towards glycolytic metabolism rather than more effective mitochondrial oxidative phosphorylation even in the presence of sufficient oxygen. This characteristic effect was later termed as the Warburg effect. One of the possible mechanisms of the Warburg effect is that the decrease in mitochondrial pyruvate oxidation was due to its import inhibition in cancer cells [13, 15, 87, 110, 111]. SIRT3^−/−^ MEF study demonstrates that cancer cells consume more glucose. They have higher levels of glycolytic intermediates and lower levels of TCA cycle intermediates and also exhibit hyperactivation of HIF-1*α* target genes which is consistent with the Warburg effect. Another mechanism of genotoxic stress and increased chromosomal instability is possible due to the higher level of ROS and superoxide radicals [10, 13, 18, 75, 76, 87].

SIRT3 overexpression in cases of several breast cancer cell lines has been able to reverse the Warburg effect [13, 15, 87, 110]. In humans, abnormally low levels of SIRT3 expression are found to be associated with lung cancer [13]. Similarly, SIRT3 was found to be associated with oral squamous cell carcinomas (OSCCs) and glioma. In a glioma cell line, in vitro overexpression of SIRT3 was protective against apoptosis and significant progression of cell cycle towards G2/M. This suggests that SIRT3 overexpression favors cell survival. SIRT3 overexpression also drastically modifies the methylation profile of a glioma-derived cell line, with the hypermethylation of numerous genes at CpG islands [112, 113].

SIRT3 deacetylates p53 in vitro like SIRT1, and it also rescues cancer cell from p53-mediated growth arrest through downregulation of Mdm2 and can potentially play an important role in the development of hepatocellular carcinoma (HCC) [114]. Similarly, Liu et al. [115] found that low-level of SIRT3 was associated with the occurrence and development of HCC. SIRT3 inhibited HepG2 via SOD2 and p53 which upregulated Bax and Fas. In a recent systemic review and meta-analysis by Zhou et al. [116], SIRT3 was found to have a diverse role in the neoplasm. SIRT3 was associated with the bad prognostic factor in BC, CC, and NSCLC, whereas it was associated with the good prognostic factor in CLL, HCC, PC, and RCC, especially in HCC.

Therefore, SIRT3 in certain cells could potentially enhance tumorigenesis, promote survival signals, and suppress apoptotic signals. And in these cases, SIRT3 overexpression resulted in the disease reoccurrence and progression that led to the poor prognosis.

## 2. Discussion and Conclusion

Hepatic IRI is characterized by sterile inflammation, hepatocellular damage, and postoperative liver dysfunction after liver resection or transplantation [117–119]. Liver damage by IRI is a global process that includes several cellular and humoral pathways [120, 121]. Mitochondria are the powerhouse of cells that generate ATP which are used up as the energy source for the vital living activity [122]. Although ROS is a harmful byproduct of mitochondrial metabolism, the new concept of mitochondrial hormesis or mitohormesis challenges this traditional concept by claiming that ROS exert a nonlinear or J-shaped response and are essential stress-signaling molecules that bring about nuclear transcriptional changes for the cellular adaptation [2]. Several recent studies have shown that mitochondrial sirtuins, particularly SIRT3 via different mechanism, regulate a wide range of cellular processes like apoptosis, aging, cellular senescence, transcriptional silencing, DNA repair, and genome stability, enhance stress resistance, and control of metabolic enzymes by the attenuation of harmful activities of ROS [21, 112, 123, 124].

Finally, to summarize, we have compiled the current evidence on how SIRT3 along with its downstream mediators (particularly SOD2, CypD, and HIF-1*α*) attenuate the mitochondrial ROS and thus preserve the function of the remnant liver following the major surgeries and the transplantation. Animal trials with drugs that modulate the activity of SIRT3 and its downstream mediators have shown promising results in hypoxic insults/ischemia-reperfusion injury, during low energy input and tumor suppression. Further research on these novel proteins shall open up better perspective in the field of hepatic IRI and liver transplantation.

## Abbreviations

ADP: Adenosine diphosphate
AMP: Adenosine monophosphate
AMPK: Adenosine monophosphate-activated protein kinase
A1R: Adenosine A1 receptor
AKI: Acute kidney injury
ANT: Adenine nucleotide translocator
ARNT: Aryl hydrocarbon nuclear translocator
AST: Aspartate aminotransferase
BAK: Bcl-2 homologous antagonist/killer
BAX: Bcl-2-like protein 4
Bcl-2: B-cell lymphoma 2
CDCA: Chenodeoxycholic acid
CR: Caloric restriction
CypD: Cyclophilin
FOXO3a: Forkhead box O3a
GSK 3*β*: Glycogen synthase kinase-3*β*
HCC: Hepatocellular carcinoma
HFD: High-fat diet
HIF: Hypoxia-induced factor
HTK: Histidine-tryptophan-ketoglutarate
IMM: Inner mitochondrial membrane
LECs: Liver epithelial cells
OMM: Outer mitochondrial membrane
IGL-1: Institute Georges Lopez preservation solution
IPC: Ischemic preconditioning
I/R, IRI: Ischemia/reperfusion injury
KA: Kainic acid
LCAD: Long-chain acyl-CoA dehydrogenase
LDH: Lactate dehydrogenase
LRP130: Leucine-rich protein 130
GPx1: Glutathione peroxidase 1
UCP2: Uncoupling protein 2
MAPK: Mitogen-activated protein kinase
MnSOD: Manganese superoxide dismutase
mPTP: Mitochondrial permeability transition pore
NAD+: Nicotinamide-adenine dinucleotide
NF-*κ*B: Nuclear factor kappa B
NO: Nitric oxide
OGG1: Oxoguanine-DNA glycosylase-1
OSTa/OSTb: Organic solute transporter a/b
OXPHOS: Oxidative phosphorylation
PHD: Prolyl hydroxylase domain
PGC1*α*: Peroxisome proliferator-activated receptor gamma coactivator 1-alpha
RSV: Resveratrol
ROS: Reactive oxygen species
SIRT: Sirtuin
SOD: Superoxide dismutase
SREBP: Sterol regulatory element-binding protein
IPC: Ischemic preconditioning
UW: University of Wisconsin solution
VDAC: Voltage-dependent anion channel.
